# M2 Macrophages-Based Immunotherapy: A New Therapeutic Approach in Liver Fibrosis

**DOI:** 10.34172/apb.025.43855

**Published:** 2025-06-02

**Authors:** Wahyu Widowati, Adilah Hafizha Nur Sabrina, Annisa Firdaus Sutendi, Fadhilah Haifa Zahiroh, Teresa Liliana Wargasetia, Ita Margaretha Nainggolan, Elham Rismani, Massoud Vosough

**Affiliations:** ^1^Faculty of Medicine, Maranatha Christian University, Bandung 40164, Indonesia; ^2^Aretha Medika Utama, Biomolecular and Biomedical Research Center, Bandung 40163, Indonesia; ^3^Eijkman Research Center for Molecular Biology, National Research and Innovation Agency, Bogor, Indonesia; ^4^School of Medicine and Health Sciences, Atma Jaya Catholic University of Indonesia, Jakarta, Indonesia; ^5^Molecular Medicine Department, Biotechnology Research Center (BRC), Pasteur Institute of Iran, Tehran, Iran; ^6^Department of Regenerative Medicine, Cell Science Research Center, Royan Institute for Stem Cell Biology and Technology, Tehran, Iran; ^7^Experimental Cancer Medicine, Institution for Laboratory Medicine, and Karolinska University Hospital, Karolinska Institute, Stockholm, Sweden; ^8^Department of Cellular and Molecular Biology, Faculty of Sciences and Advanced Technology in Biology, University of Science and Culture, Tehran, Iran

**Keywords:** Anti-inflammatory, Immunotherapy, Liver fibrosis, M2 macrophages, Tissue repair

## Abstract

Liver fibrosis (LF) is a pathological condition resulting from a chronic inflammatory response to multiple etiological factors, including viral infections, excessive alcohol consumption, and metabolic disorders. The important role of macrophages in this process, especially the M2 subtype, has attracted attention as a potential target for macrophage-based immunotherapy. M2 macrophages have anti-inflammatory and reparative properties that enable them to modulate the immune response and facilitate repairing damaged tissues. They participate in reducing fibrogenic features in term of gene expression and histological markers associated with LF. These cells phagocytose apoptotic cells and matrix components. M2 macrophage-based immunotherapy has shown great potential in ameliorating LF through mechanisms involving the IL-10/STAT3 and TGF-β/SMAD signaling pathways, which are essential in suppressing the pro-inflammatory response and supporting tissue regeneration. However, significant challenges such as individual resistance to therapy and the potential for promoting fibrosis suggest that further development and research are needed to optimize the safety and efficacy of this therapy in clinical applications. This study provides comprehensive insights into the role of M2 macrophages in LF and explores their potential as an innovative therapeutic approach in treating LF.

## Introduction

 The liver serves as a vital center for numerous physiological processes, including the metabolism of macronutrients, regulation of blood volume, immune system support, endocrine control of growth, signaling pathways, homeostasis of lipid and cholesterol, and the detoxification of xenobiotic compounds, including many existing drugs.^1^ Liver diseases form a heterogeneous group of acute and chronic disorders with various etiologies, including drug-induced liver injury, acute-on-chronic liver failure, and non-alcoholic fatty liver disease, with a predominance of viral hepatitis and cancerous state.^2^ Acute liver failure (ALF) is an uncommon medical condition characterized by rapid decline in liver function, leading to significant deterioration and coagulopathy in patients without a prior history of liver disorders, which often impacts young people and is associated with considerable morbidity and mortality.^3^ In developed countries, the primary factor contributing to ALF is liver damage caused by pharmaceuticals, most commonly by paracetamol. Other causes are acute viral infections of hepatitis A, B, and E.^4^

 ALF causes a recurrent inflammatory response that triggers the production of liver fibrosis (LF). Over time, fibrosis can progress to cirrhosis, the advanced stage of ALF. Cirrhosis is a significant precancerous condition for hepatocellular carcinoma (HCC). The typical effect of all these disorders on the liver is the emergence of chronic inflammation, resulting in an abnormal wound-healing response.^5^

 Given the liver’s vulnerability to a range of diseases, understanding the involvement of liver macrophages in the inflammatory response becomes crucial for developing effective therapeutic strategies against LF. Liver macrophages are vital members of the mononuclear phagocyte system, playing a central role in inflammation-related liver disorders due to their ability to respond to diverse activating signals.^6^ Macrophages in the liver are categorized into two different phenotypes: M1 and M2. The M1 phenotype is distinguished by its elevated synthesis of pro-inflammatory cytokines, heightened levels of reactive nitrogen and oxygen species, initiation of Th1 responses, and potent microbicidal and tumoricidal capabilities. M2 macrophages are crucial in controlling parasite infections, facilitating tissue restructuring and tumor progression, and regulating immunological reactions.^7^ The activation of M2-phenotype macrophages releases profibrotic cytokines, which contribute to LF through the interleukin (IL)-13 receptor alpha 1 pathway.^8^ The prevalence of M2-like macrophages in LF, together with their profibrotic functions, and the ability to modify their polarization and activity, provide a compelling rationale for targeting M2 macrophages as a therapeutic strategy in LF. Therefore, this study is to provide an understanding of M2 macrophage-based immunotherapy and its potential as a therapeutic approach in LF. This objective is achieved by discussing LF pathogenesis and treatment, macrophages in LF, M2 macrophage functions and characteristics, M2 macrophages in LF, preclinical evidence, therapeutic approaches, the benefits of targeting M2 macrophages in LF therapy, and future perspectives based on the information obtained. The scope of the information is limited to the liver, LF, M2 macrophages, and M2 macrophages as a therapeutic approach in LF.

## Liver fibrosis: pathogenesis and current therapy

**Figure 1:**
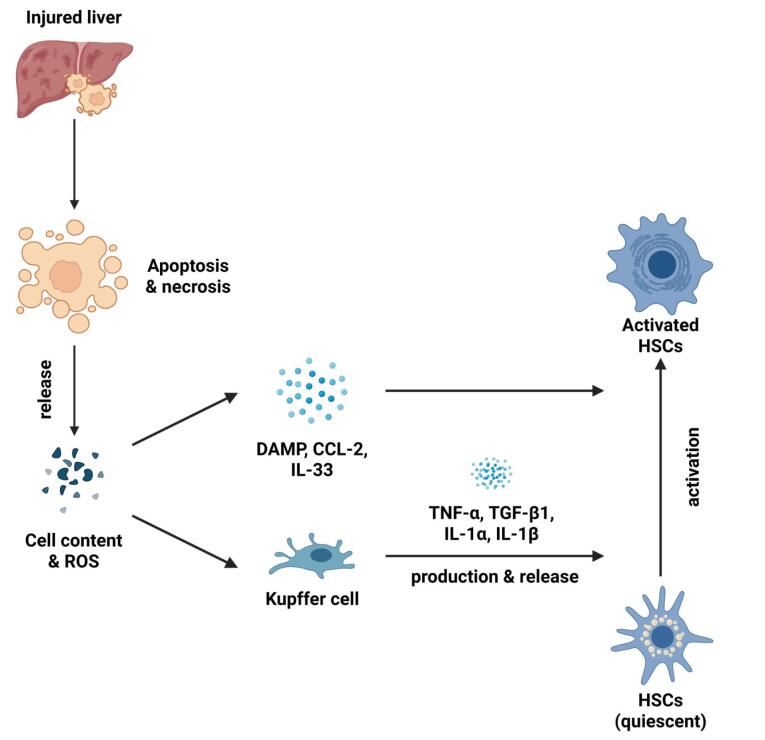
Mechanism of HSCs activation.The sequence of events in liver injury leading to fibrosis begins with hepatotoxic signals that induce necrosis or apoptosis of hepatocytes, leading to the release of cellular contents and ROS. The release of damage-associated molecular patterns (DAMPs) and signaling molecules such as C-C motif chemokine ligand 2 (CCL2) and IL-33 is a consequence of this cellular damage. Simultaneously, macrophages are also activated, releasing pro-inflammatory cytokines (TNF-α, TGF-β1, IL-1α, IL-1β) that activate HSCs. Activated HSCs produce collagen and extracellular matrix components, which contribute to the formation of liver fibrosis

 LF is a condition that arises because of the activation of the hepatic stellate cells (HSCs) as shown in Figure 1. HSCs are localized perisinusoidal cells found in the subendothelial region situated between hepatocytes and sinusoidal endothelial cells.^9^ This area, filled with permeable connective tissue, facilitates the exchange of biomolecules between portal blood and hepatocytes.^10^ In response to liver injury, quiescent HSCs proliferate and trans-differentiate into contractile myofibroblasts. This process is triggered by paracrine signals from neighboring cells, including Kupffer cells, hepatocytes, platelets, leukocytes, and sinusoidal endothelial cells. Kupffer cells, in particular, drive HSCs activation and proliferation through the release of cytokines such as TGF-β1, IL-1, tumor necrosis factor (TNF), reactive oxygen species (ROS), and lipid peroxides.^11^ TGF-β1 is a central cytokine in HSC activation and is a major mediator of LF.^12^

 Recently, there have been many LF therapy variations, such as drug therapy, cell therapy, and liver transplantation and extension, as shown in Table 1.

**Table 1:** Current treatment for liver fibrosis

**Therapy**	**Objective**	**Mechanism**	**Reference**
**Drug therapy**			
Sofosbuvir	Primarily for hepatitis C treatment	Reduces inflammation and fibrosis	^13,14^
Ledipasvir	Approved for hepatitis C, often combined with sofosbuvir	Inhibits HCV replication	^13^
Lutathera (Lutetium Lu 177 Dotatate)	Approved for neuroendocrine tumors	Helps reduce tumor-related fibrosis	^15^
Obeticholic Acid	Approved for NASH and fibrosis	Farnesoid X receptor (FXR) agonist; reduces bile acid synthesis and inflammation, impacting fibrosis progression	^16^
Pirfenidone	Approved for idiopathic pulmonary fibrosis (IPF); in trials for LF	Inhibits fibrosis and inflammation; originally used for IPF).	^17^
Nintedanib	Approved for IPF; in trials for LF	Tyrosine kinase inhibitor; target multiple pathways involved in fibrosis	^18^
Cenicriviroc (CVC)	In clinical trials for NASH	CCR2/CCR5 antagonist; reduces inflammation and fibrosis by blocking specific receptors	^19^
Resmetirom (MGL-3196)	In clinical trials for NASH	Thyroid hormone receptor beta agonists aim to reduce liver fat and fibrosis.	^20^
**Cell therapy**			
Endothelial progenitor cells (EPCs)	Restore liver function and increase survival rate	Suppress HSCs reduce levels of aspartate aminotransferase (AST) and alanine aminotransferase (ALT) and enhance hepatocyte proliferation and expression of hepatocyte growth factor (HGF) and vascular endothelial growth factor (VEGF) in serum	^21^
Bone marrow mononuclear cells (BMMNCs)	Improve mitochondrial bioenergetics	Stimulate liver oxidative capability, reduce oxidative stress, and regulates mitochondrial coupling and biogenesis.	^22,23^
Bone marrow mesenchymal stem cells (BMMSs)	Reduce collagen, induce HSCs apoptosis, reduce pro-inflammatory cytokines, and aid liver enzyme recovery.	Decrease collagen deposition, promote HSCs apoptosis, and improve liver enzyme levels.	
Adipose-derived mesenchymal stem cells (ADSs)	Inhibit the activation and proliferation of HSCs and reduce AST/ALT levels.	More effective in preventing HSCs activation and proliferation and lowering AST/ALT levels.	
Wharton’s Jelly mesenchymal stem cells (WJ-MSC) Extracellular vesicle (EV)	Guiding macrophages toward an anti-inflammatory immunophenotype	EVs induce activated macrophages to modulate immune responses, potentially contributing to a protective function in LF pathogenesis by directly inhibiting the activation of HSCs	^24^
Human amniotic epithelial cells (hAECs)	Inhibit HSCs activation	LF is reduced by inhibiting TGF-β and PDGF signaling pathway while enhancing the secretion of anti-fibrotic factors such as PGE-2, BMP-7, and IL-10. It also reduces ECM deposition and revert the myofibroblast phenotype to fibroblast.	^25^
**Transplantation**			
Liver transplantation	Only life-saving option for advanced LF	Replaces a diseased liver with a healthy one, thereby restoring normal liver function.	^22^

## M2 macrophages: Characteristics and functions

 The liver houses ninety percent of the body’s macrophages, which originate from various sources, leading to significant cellular diversity. This diversity results in varied cytokine production, cell surface markers, and transcriptional profiles. Macrophages are essential immune cells in various inflammatory processes and tissue healing, particularly in the context of LF. Several studies have shown that macrophages in tissues, including the liver, exhibit ontogenetic heterogeneity, originating from both embryonic sources, such as Kupffer cells, and from bone marrow-derived monocytes. These two origins play distinct roles in regulating the development and resolution of LF, leading to different macrophage subsets having varying effects on LF.^26^ Migration and transformation of hepatic macrophages happen based on the microenvironmental signals, allowing them to adopt pro-inflammatory (M1) or anti-inflammatory (M2) roles.^27^

**Figure 2:**
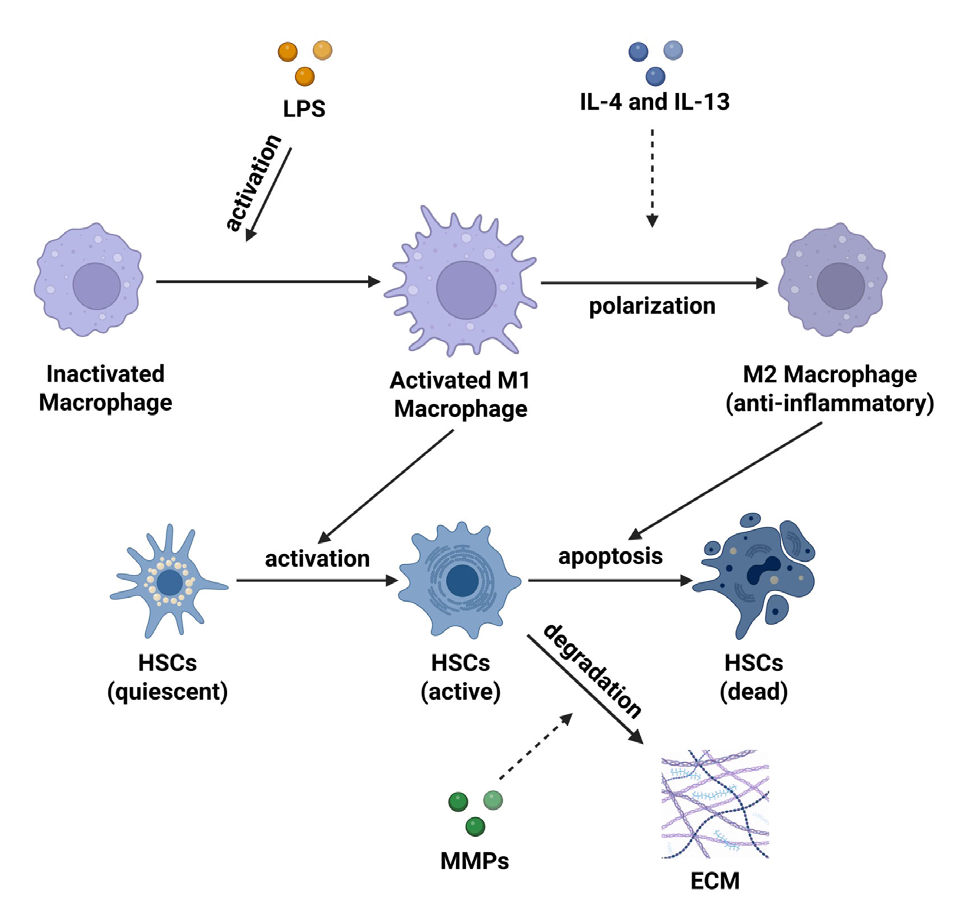
Molecular mechanisms of M2 macrophages in LF. Pro-inflammatory M1 macrophages are activated by lipopolysaccharide (LPS) and activate HSCs in an active form contributing to fibrosis. The cytokines IL-4 and IL-13 polarize M1 into anti-inflammatory M2 macrophages, which induce HSCs degradation through apoptosis. M2 macrophages also release matrix metalloproteinase (MMPs) to degrade ECM and reduce fibrosis

 M2 macrophages, also known as alternatively activated macrophages, represent a specific subtype of macrophages that are involved in immune regulation, tissue repair, and inflammation resolution (Figure 2). Primarily, this phenotype is activated by IL-4 and IL-13 produced by T helper 2 (Th2) cells,^28^ and typically identified by their notable expression of functional surface marker such as CD163.^29^ Nevertheless, macrophage malfunction can hinder the normal tissue repair process and conversely, facilitate the occurrence of fibrosis, the accumulation of type I and III collagen, and the activation of myofibroblasts.^30^ An excessive amount of M2 macrophages is associated not only with fibrosis, but also with cancer,^31^ chronic obstructive pulmonary disease (COPD),^29^ and acute kidney injury.^32^

 Research into the role of M2 macrophages in LF is ongoing, but early findings suggest that targeting M2 macrophages could represent a viable therapeutic approach for managing LF (Table 2). Therapeutic agents can be used to increase the number of M2 macrophages in the liver, which may help reduce inflammation and promote tissue repair, enhancing M2 macrophage activity.^33^

**Table 2:** Studies on the role of M2 macrophages in liver fibrosis

**Model**	**Description**	**Role of M2 Macrophages**	**References**
Carbon tetrachloride (CCl4)	CCL4-induced mice as a LF model.	M2 macrophages promote fibrogenesis by producing TGF-β to activate HSCs, increasing extracellular matrix (ECM) deposition, and facilitating resolution of LF through matrix metalloproteinases (MMPs).	^34^
Bile duct ligation (BDL)	This rat model involves bile duct obstruction, leading to cholestasis and LF. This model allows the study of the initiation and resolution phases of LF.	M2 macrophages are activated by profibrotic cytokines and growth factors, promoting the deposition of collagen and ECM components that contribute to LF development. In the resolution phase, M2 is activated by pro-inflammatory signals to switch from profibrotic to antifibrotic function, promoting the degradation of collagen and other fibrotic components.	^35^
Diet-induced models	A high-fat diet is used to induce non-alcoholic fatty liver disease (NAFLD) in rats, which is characterized by steatosis, inflammation, and LF. This model is useful for studying LF among others.	In the initiation phase, M2 macrophages are activated by profibrotic cytokines and growth factors, promoting the deposition of collagen and ECM components. In the resolution phase, M2 contributes to the degradation of collagen and other fibrotic components.	^36^
Intraperitoneally with 3 μl/g (30%) CCI4	BALB/c mice were injected with a lethal dose of hepatic toxin: Intraperitoneally with 3 μl/g (30%) CCI4 twice a week for 6 weeks to induce LF and acute liver injury	M2 macrophages resulted in a notable decrease in mRNA levels of injury mediatory (TNF-α, IL-1β, IL-6, IL-12, high mobility group box 1 (HMGB1), and MMP-9), pro-inflammatory cytokines (IL-12, IL-17, and TNF-α), and developed apoptosis resistance in hepatocyte	^37^
Intraperitoneally (i.p) with 0.75 ml/kg of CCl4	Intraperitoneally (i.p) injection of male mice to induce LF	Kupffer cell expanded *in vitro* had the potential of M1/M2 polarization of macrophage and can reduce the alpha-smooth muscle actin (αSMA)-positive signals, which means the reduction of HSCSs and decreased IL-1β, IL-6, and TNF-α level	^38^
Acetaminophen (APAP)	8-week-old mice were injected APAP in warm sterile saline to promote necrosis, acute liver injury, and LF	Macrophages rapidly mitigated liver damage and diminished various inflammatory mediators.	^39^

## M2 macrophages in liver fibrosis: Insights from experimental and clinical research

 Multiple preclinical and clinical investigations have examined the involvement of M2 macrophages in the progression of LF. Research conducted in laboratory settings and on patients indicates that M2 macrophages are crucial function in regulating the inflammatory milieu, fibroblast activity, and resolving fibrosis. The findings of these investigations are presented in Table 3, which demonstrates the impact of M2 macrophages on the process of fibrogenesis.

**Table 3:** Preclinical and clinical studies about M2 macrophages inLF

**Category**	**Description**	**References**
In vitro	IL-4 and IL-13 can induce M2 macrophages to produce an anti-inflammatory environment that can promote fibrogenesis through the secretion of the profibrotic factor TGF-β.	^40-42^
In vitro	Kupffer cells inhibit T cell response when induced with regulatory T cells	^43^
In vivo	M2-polarized macrophages protect hepatocytes against cell death, suggesting their role in providing protective effects against severe damage or lethal insults in the liver.	^37^
In vivo	M2 macrophages are capable of secreting cytokines like IL-10 and expressing signature molecules such as mannose receptors, which are commonly linked to the suppression of inflammation and the promotion of tissue repair.	^44^
In vivo	M2 macrophages depletion can reduce collagen accumulation and activate fibroblasts in LF, suggesting significant role in the progression of the disease	^41,42^
In vivo	Granulocyte-macrophage colony-stimulating factor (GM-CSF) improves the purity, expression, and proliferation of Kupffer cells, and has the potential of M1/M2 polarization and phagocytosis, which can reduce the αSMA-positive signals, which means the reduction of HSCSs. and decreased IL-1β, IL-6, and TNF-α levels.	^38^
Clinical	M2 can inhibit TGF-β production, reversing hepatocellular senescence and promoting fibrosis resolution.	^42,45-47^

## Therapeutic approaches targeting M2 macrophages in liver fibrosis

 The most effective method of preventing fibrosis is to address the underlying causes, such as discontinuing alcohol consumption in patients with alcoholic liver disease. Nevertheless, this approach is not viable when other factors precipitate LF. Current management of LF primarily focuses on preventing further damage, reducing inflammation from viral infections, the suppression of HSCs activation and proliferation, and promoting the breakdown of excessive ECM in the liver.^48,49^ However, these methods have had limited success in reversing established fibrosis. Emerging evidence suggests that targeting macrophages, particularly the pro-fibrotic M2 phenotype, may offer a promising therapeutic approach for LF. Although the mechanisms and therapeutic potential of M2 macrophages in LF are being actively investigated, a comprehensive understanding of their role in disease pathogenesis is still needed to fully exploit this target for effective treatment strategies.

 Macrophages exhibit remarkable plasticity, capable of adopting diverse phenotypes in response to the signals they receive from their surrounding environment. Given the contrasting roles of M1 and M2 phenotypes, a promising therapeutic approach is to shift macrophages from pathogenic to restorative phenotypes. However, this approach presents unique challenges, as excessive polarization towards the M2 phenotype can exacerbate fibrosis by stimulating collagen production and myofibroblast development.^50^ Therefore, therapeutic interventions should focus on reducing the accumulation and activation of M2 macrophages in the liver. Studies in other fibrotic diseases, such as idiopathic pulmonary fibrosis (IPF) and acute respiratory distress syndrome (ARDS)-associated fibrosis, have highlighted the critical role of M2 macrophages in driving excessive ECM deposition and impaired tissue repair.^51,52^

 Another potential strategy is the selective depletion of M2 macrophages from the disease microenvironment. This can be achieved using agents that specifically target and induce apoptosis or depletion of M2 macrophages, such as clodronate-containing liposomes or antibodies targeting M2-specific surface molecules like CD163 or CD206.^53^ Additionally, innovative CAR-T cell-based therapies are being developed to recognize M2 macrophage-specific antigens, particularly in oncology applications.^54^ In the context of LF, depleting M2 macrophages from the liver microenvironment can reduce their contribution to uncontrolled fibrotic processes, thereby slowing the progression of LF.^27^ This approach not only targets the fibrogenic activity of M2 macrophages but also holds potential for enhancing overall liver health and function.

 Beyond depletion, reprogramming M2 macrophages into M1 macrophages offers a promising strategy to shift the liver microenvironment dynamics from a profibrotic to a pro-resolution state. M1 macrophages contribute in enhancing the immune response, facilitating tissue repair, promoting collagen degradation, and resolving inflammation, all of which can help prevent or mitigate the progression of LF. During LF progression, M2 macrophages release various profibrotic mediators, such as TGF-β and arginase-1, which exacerbate fibrosis conditions. Targeting these mediators can disrupt the molecular pathways driving fibrosis, thereby reducing profibrotic stimulation, and slowing disease progression. Ying and colleagues’ study demonstrated that inhibiting TGF-β secreted by M2 macrophages effectively reduced HSC activation and collagen deposition.^55^ This approach not only addresses the immediate fibrotic response but also promotes a healthier liver microenvironment conducive to regeneration.

 Overall, therapeutic strategies targeting M2 macrophages hold the potential to effectively intervene in LF progression either reducing profibrotic activity or promoting fibrosis resolution. Modulating the quantity, function, or secretory products of M2 macrophages is expected to improve the immunological and fibrogenic balance within the liver, ultimately slowing or even reversing LF progression. However, further research is essential to fully realize their therapeutic potential and develop more effective interventions for managing LF. Continued exploration in this area could pave the way for innovative treatments that enhance liver health and improve patient outcomes.

## Rationale for targeting M2 macrophages in LF treatment

 M2 macrophages represent a great potential for immunotherapy in LF due to their unique capabilities in modulating immune responses and promoting tissue repair. Recent studies highlight several advantages of utilizing M2 macrophages, particularly those derived from human induced pluripotent stem cells (iPSCs). These M2 macrophages have been shown to significantly downregulate fibrogenic gene expression and histological markers associated with LF.^56^ Furthermore, M2 macrophages are key players in modifying the IL-10/TGF-β signaling pathway, which has therapeutic implications beyond LF. For instance, their involvement in alleviating Adriamycin nephrosis—a kidney disorder—demonstrates their ability to suppress inflammatory responses and facilitate tissue repair. This mechanism suggests that similar strategies could be applied to enhance liver regeneration and recovery in LF patients.^57^

 M2 macrophages are also crucial in fibrosis resolution by engaging in phagocytosis of apoptotic cells and ECM components, which facilitates tissue remodeling. They are also known to enhance fibroblast proliferation and collagen synthesis, thereby contributing to the healing process.^58,59^ Furthermore, M2 macrophages express arginase-1, an enzyme that is associated with suppression of fibrotic responses, further emphasizing their protective role in tissue repair.^60^ The important role of M2 macrophages in wound-healing is also seen through the release of anti-inflammatory cytokines and micronutrient management to support tissue repair. However, in the context of chronic infection, M2 macrophages can paradoxically contribute to the development of tissue fibrosis and cancer while also to suppress Th1 immune responses.^61^ Supporting this duality, M2 macrophages have been shown to reduce ROS production and secrete a variety of anti-inflammatory factors, including IL-4, IL-10, and insulin-like growth factor-1 (IGF-1). These factors not only promote tissue repair but also aid in the clearance of cellular debris and stimulate regenerative processes.^62^ Thus, while M2 macrophages are essential for resolving fibrosis and promoting healing, their roles must be carefully considered within the broader context of chronic inflammatory diseases.

 More specifically, M2 macrophages are involved in several critical signaling pathways that regulate inflammation and tissue repair. One key mechanism is the release of IL-10 by M2 macrophages, which activates the STAT3 signaling pathway. This activation is pivotal for the suppression of pro-inflammatory cytokines, thereby mitigating chronic inflammation and inhibiting the activation of HSCs. By doing so, IL-10 helps reduce collagen production and fibrosis, illustrating the anti-fibrotic potential of M2 macrophages.^61^ STAT3 is a central convergence point of various signaling pathways involved in inflammation, immunity, fibrosis, and oncogenesis. It functions as a key regulator of various macrophage biological activities.^63^ Additionally, M2 macrophages activate the TGF-β pathway, which engages the Sma and Mad-related protein (SMAD) signaling cascade. This pathway not only supports tissue regeneration but also inhibits excessive inflammatory responses. Although TGF-β can promote fibrosis, its activation by M2 macrophages strikes a crucial balance between facilitating tissue repair and preventing HSCs activation. Activation of the TGF-β/SMAD pathway increases the expression of BW-derived M2 macrophage markers and decreases M1 macrophage markers. This shift promotes wound closure and tissue healing, as demonstrated in studies involving diabetic mice.^64^ In summary, the interplay between IL-10 and TGF-β signaling pathways highlights the reparative functions of M2 macrophages, positioning them as essential players in managing fibrosis and promoting tissue regeneration.

 Other signaling pathways that underscore the role of M2 macrophages in promoting tissue repair and modulating inflammation include phosphoinositide 3-kinase (P13K)/ protein kinase B (AKT), which is activated by IL-4 and IL-3 receptors on M2 macrophages. This activation supports cell survival and enhances anti-inflammatory activities, thereby facilitating wound healing and liver regeneration. Notably, the PI3K/AKT pathway plays a crucial role during inflammation by converting microglia from a pro-inflammatory M1 phenotype to an anti-inflammatory M2 phenotype. This transition results in increased expression of anti-inflammatory cytokines such as IL-1 receptor antagonist (IL-1ra), IL-10, and interferon-beta, while simultaneously decreasing levels of pro-inflammatory cytokines like IL-1, TNF-α, IL-6, IL-8, and C-X-C motif chemokine ligand (CXCL1).^65,66^ Furthermore, M2 macrophages promote apoptosis, contributing to the resolution of inflammation.^67^ Another critical pathway is the signaling pathway of peroxisome proliferator-activated receptor gamma (PPAR-γ). Activation of PPAR-γ in M2 macrophages increases the expression of anti-inflammatory genes, reduces inflammation, reduces overall inflammation, and mitigates fat accumulation in the liver, which collectively helps to decrease fibrosis. This pathway also plays a protective role by preventing excessive activation of profibrotic pathways such as TGF-β.^68^ In addition, the IL-4/IL-13/ signaling pathway through signal transducer and activator of transcription 6 (STAT6) promotes the expression of reparative and anti-inflammatory genes in M2 macrophages. This signaling cascade not only reduces inflammation but also aids in liver tissue repair.^69^ Finally, the nuclear factor kappa-light-enhancer of activated B cells (NF-kB) signaling pathway activated by M2 macrophages can help suppress chronic inflammation while supporting tissue resolution and repair processes.^70,71^ In summary, these diverse signaling pathways illustrate the multifaceted roles of M2 macrophages in regulating inflammation and promoting tissue regeneration, highlighting their potential as therapeutic targets in managing LF and other related conditions.

## Challenges of M2 macrophage-based immunotherapy

 Despite promising potential of M2 macrophage-based immunotherapy for the treatment of LF, it also presents several notable challenges that must be addressed for successful clinical application. One major concern is the context-dependent behavior of macrophages; studies using animal models have shown that macrophages can have different, or even opposite, roles depending on the experimental conditions. Therefore, careful consideration of factors such as dosage, timing of intervention, and macrophage subsets to be targeted according to the stage of the disease. In addition, the mouse models employed in this research did not completely reflect the complex disease conditions in humans. These models typically reflect only specific pathological processes induced by certain stimuli, thereby neglecting the multifaceted nature of LF as it occurs in human patients.^72^

 Different factors contributing to LF also cause different pathogenesis and pathological processes, thus requiring different effects of macrophage polarization. For example, studies have shown that M1 macrophages and their associated pro-inflammatory cytokines are significantly increased in LF induced by CCl4. In contrast, M2 macrophage polarization appears to be more dominant in LF resulting from schistosomiasis infection.^73^ In addition, M2 macrophages are known for their profibrotic properties, producing growth factors and ECM components that contribute to scar tissue formation. They also secrete proteases that help break down ECM, thereby aiding tissue repair. However, if these processes are not tightly regulated, they can potentially promote tumorigenesis.^74^ This highlights the complexity of macrophage roles in LF and underscores the need for tailored therapeutic strategies that consider the specific context of macrophage polarization in different etiologies of LF.

 The response to M2 macrophage-based immunotherapy can also vary among individuals, depending on factors such as genetics, existing health conditions, and the extent of fibrosis.^75^ In some cases, LF may show resistance to M2 macrophage therapy, mainly if significant pathological changes have already occurred, potentially limiting the long-term effectiveness of this approach. Furthermore, although M2 macrophages can inhibit the activation of HSCs, improper regulation can lead to excessive collagen production, exacerbating fibrosis rather than alleviating it. Directing M2 macrophages to specific areas of the liver affected by fibrosis is also challenging, as without proper targeting, M2 macrophages may not be able to reach the desired location or even cause side effects. Furthermore, an imbalance in the M1/M2 ratio leads to HSCs activation and LF development.^61^ This complexity underscores the need for a nuanced understanding of individual patient profiles and the intricate dynamics of macrophage polarization in order to optimize the effectiveness of M2 macrophage-based therapies for LF.

 Overall, M2 macrophage-based immunotherapy approaches offer great potential in treating LF; however, the efficacy of these therapies highly relies on addressing the challenges that exist. Further research and development of more sophisticated technologies are needed to guarantee these therapies in clinical applications.

## Future perspective and research direction

 The potential of utilizing M2 macrophage-based immunotherapy in treating LF shows promise. However, various areas need further investigation to optimize therapeutic techniques and enhance patient outcomes. Subsequently, investigations should prioritize the subsequent critical avenues:

###  Optimisation of targeting strategies

 Non-targeted medicines can impact on several cell types, including M1 macrophages, other immune cells, and healthy cells in different organs. This broad impact can result in undesirable side effects, such as systemic inflammation or compromised immunological function. For example, anti-fibrotic drugs such as interferon-gamma (IFN-γ), angiotensin II, and interleukin 10 have shown promising results in preclinical trials; however, they have not translated successfully into clinical settings.^76^ The primary reason for these failures is the lack of specific delivery mechanisms in their formulations. While IFN-γ possesses recognized anti-fibrotic properties, it also induces pro-inflammatory responses in macrophages, complicating its therapeutic use.^77^ One promising avenue to tackle this issue is the application of nanomedicine. Nanomedicines enable precise control over drug distribution, preventing premature drug breakdown and improving drug absorption. Nanoparticles have the potential to enhance biocompatibility and stability of therapeutic agents in comparison to traditional medications. This targeted approach could optimize the efficacy of anti-fibrotic therapies while mitigating adverse effects, paving the way for more successful treatment strategies in LF management.

 Furthermore, nanoparticles can be precisely designed to provide focused therapeutic benefits.^78^ To improve the specificity, active targeting approaches can be implemented by modifying the surface of the nanoparticles to establish a selective affinity for recognizing and interacting with specific receptors on the surface of macrophages, including mannose, dectin-1, Tuftsin peptide, folate receptor beta (FR-b), and phosphatidylserine.^79^ A study by Singh et al has successfully engineered mannosylated albumin nanoparticles (MANPs) that specifically target CD206 + macrophages in pulmonary fibrosis.^80^ By delivering small-interfering RNAs (siRNA) via the MANPs pathway, these nanoparticles can silence the TGF-β1 signal. This approach is also found in a study by Tran et al. M2 polarization was successfully induced by delivering microRNA-223 (miR-223) using special nanoparticles.^81^ These nanoparticles are designed to target CD44 and carry miR-223 duplexes or plasmid DNA that expresses miR-223, which helps repolarize the macrophages.

 The therapeutic mechanisms of nanoparticles and liposomes in treating LFs involve several pathways, including inhibition of TGF-β production, reduction of collagen deposition, and modulation of M2 macrophage function. For example, silica nanoparticles have demonstrated the ability to inhibit collagen production and reduce LF in preclinical models. In contrast, gold nanoparticles have been used to deliver siRNA to the liver and reduce LF. Lipid-coated nanoparticles have been designed to target M2 macrophages through specific receptors, facilitating the delivery of therapeutic compounds to the disease. Polymeric nanoparticles have also been employed to transport therapeutic agents to the liver and have shown promising results in reducing LF.^82^ Besides that, other nanoparticles like silicon dioxide (SiO_2 _NPs) and titanium dioxide (TiO_2_ NPs) are proven to inhibit the expression of collagen type 1 by upregulating matrix metalloproteinases (MMPs) and downregulating tissue inhibitors of metalloproteins (TIMPs).^83^

 Another modified engineered liposome was also found to successfully target C-X-C motif chemokine receptor 4 (CXCR4), which is overexpressed in idiopathic pulmonary and LF, leading to the polarization of M1/M2 macrophages. The liposomes were modified to carry an anti-fibrotic medicine with a particular MMP-2-responsive peptide called E5. This modified liposome will find and stick to cells with too much C-X-C motif chemokine ligand 4 (CXCL4). The excessive MMP-2 in fibrosis will further help the liposome break apart and release the medicine exactly where it is needed.^8^ The proportion of M1 and M2 in the fibrosis site is nearly equal after the induction of this liposome, resulting in improved antifibrotic effects.

###  Mechanistic insight and identification of novel biomarkers

 Developing a more comprehensive understanding of the mechanism and novel biomarkers that affect M2 macrophages is essential to optimize therapeutic targets. Given the intricate function of M2 macrophages, it is essential to comprehend their unique inflammatory processes in the context of LF. The research should identify how these pathways can be manipulated to optimize the therapeutic potential of M2 macrophages while simultaneously reducing the likelihood of profibrotic or tumorigenic effects.

 Originally, M2 macrophages were marked by their response to IL-4 or IL-13, as these signals are typically associated with the immune system’s reaction to parasites or fungi, triggering a Th2 immune response. However, these markers can also appear when other types of immune cells are exposed to IL-4 or IL-13, indicating that they are not exclusive to M2 macrophages.^84^ Depending on what triggers the activation, Mantovani and colleagues divided M2 macrophages into different subtypes: M2a (activated by IL-4 and IL-13) and M2c (activated by IL-10 and GCs), each having slightly different roles and markers.^85^ Mosser also added the term M2b, which refers to activation induced by Fc receptors, immune complexes, and LPS.^86^ This activation contributes to the diverse functions of M2 macrophages, including fighting viruses, producing neurotransmitters and hormones, and generating lipid mediators, which play roles in inflammation and other processes.

 Future research should focus on discovering new biomarkers for M2 macrophages and their involvement in LF, beyond the extensively studied markers. In addition to the markers mentioned above, several others are recognized as indicators of M2 macrophages, namely arginase-1 and CD206. Arginase-1 can reduce arginine availability for nitric oxide (NO) production and suppress inflammatory responses. However, in chronic inflammatory settings like fibrosis, arginase-1 will be activated by TGF-β and promote collagen production, contributing to disease progression rather than the resolution of inflammation.^87^ Thus, arginase-1 can be used as a biomarker for diagnosing and monitoring of fibrotic diseases.

 Another prognostic indicator that can be used to diagnosis acute fibrosis is CD206. CD206 is a valid marker for M2 because of its strong correlation with CD163, a marker usually used to identify the M2 phenotype.^88^ CD163 and CD206 are important receptors on the surface of macrophages. In normal conditions, macrophages release these receptors into the bloodstream to manage the inflammatory response. During ALF, the upregulation of these markers may correlate with the severity of liver disease help predict the risk of complications and monitor ALF.^89^

 Researchers can better utilize these markers in clinical settings by identifying and validating more novel biomarkers. These can enhance their utility in predicting disease outcomes and tailoring treatments, ultimately improving patient management.

###  Macrophage plasticity 

 Macrophage plasticity is the ability of macrophages to change from one phenotype to another based on the microenvironmental signals they receive.^90^ Macrophage plasticity, especially the ability of M2 macrophages to switch between different activation states, is both a challenge and an opportunity in treating of LF.^72^ Studies have shown that macrophages are capable of altering their phenotype in reaction to various environmental signals, such as cytokines, growth factors, and ECM components. This response is vital in various pathological contexts, including cancer and chronic inflammatory diseases, where macrophages can play a role in either promoting or suppressing the disease process, depending on the signal. This suggests that macrophages have high adaptability and can change dynamic phenotypes in response to environmental changes.^91^

 Cytokines have long been recognized for their ability to modulate macrophage responses, with early studies highlighting this relationship.^92^ It has been hypothesized that macrophages exhibit inflammatory or anti-inflammatory functions that corresponding to Th1- and Th2-driven immune responses, respectively.^85,93,94^ M2 macrophages acquire anti-inflammatory and pro-regenerative characteristics when are stimulated by cytokines such as IL-4 or IL-13. Specifically, IL-4 induces a distinct macrophage phenotype marked by increased mannose receptor expression and functions are markedly different from those triggered by IFN-γ.^95^ However, studies indicates that when macrophages are treated with IL-4 before LPS stimulation, they exhibit heightened inflammatory activity, including increased level of TNF-α, IL-12, and upregulation of enzymes like inducible nitric oxide synthase (iNOS) and cyclooxygenase-2 (COX-2), while simultaneously suppressing anti-inflammatory responses such as IL-10 production.^91^ This indicates that, although IL-4 is typically associated with M2 polarization, it may enhance macrophage inflammatory responses under certain conditions, such as subsequent LPS exposure. Moreover, if M2 macrophages are exposed to pro-inflammatory signals like IFN-γ or LPS, they can revert to an M1 phenotype, potentially exacerbating fibrosis. The inflammatory and cytotoxic functions of macrophages are further intensified by IFN-γ, supporting a two-signal model for activating of macrophage inflammatory responses.^92^

 Other studies have attempted to understand the mechanisms underlying macrophage plasticity to develop more effective therapeutic strategies. One approach is targeting specific signaling pathways to maintain the M2 phenotype. The IL-10/STAT3 signaling pathway has been identified as key to maintaining the M2 phenotype. The IL-10/STAT3 signaling pathway is vital in resolving inflammation and maintaining of homeostasis,^96^ by inhibition pro-inflammatory pathways such as NF-κB. In pathological contexts such as cancer or chronic infectious diseases, IL-10/STAT3 can reduce excessive inflammation on the one hand. However, it can also support a tolerogenic or immunosuppressive environment that may favor tumor growth or pathogen development. Other studies have highlighted the importance of macrophage metabolism in determining its phenotype. M2 macrophages use oxidative metabolism, which relies on oxidative phosphorylation (OXPHOS), while M1 macrophages rely more on glycolysis. M2 macrophages tend to increase fatty acid oxidation (FAO) and the activity of enzymes that support oxidative phosphorylation, allowing them to support the healing and tissue repair process.^97,98^ Therefore, manipulation of metabolic pathways may be one strategy to prevent the shift of M2 macrophages to a pro-inflammatory phenotype.

 Future research should focus on controlling macrophage plasticity to ensure that M2 macrophages remain in a therapeutic state and do not revert to a pro-inflammatory or profibrotic phenotype. Future research should focus on developing tools that dynamically monitor and control the phenotypic status of macrophages in the body. This could involve developing strategies to stabilize the M2 phenotype or selectively reprogram macrophages in the body. Future strategies may include developing gene therapies or small molecules that can stabilize the M2 phenotype, or even clustered regularly interspaced short palindromic repeats (CRISPR)-based technologies to modify gene expression in M2 macrophages to keep them in a therapeutic state. Another approach is to create a microenvironment that supports M2 macrophage activity and suppresses signals that could trigger the transition to a pro-inflammatory or profibrotic phenotype.

###  Combination therapies

 To enhance the effectiveness of M2 macrophage-based immunotherapy, future studies should prioritize the development of combination therapies. By combining the regulation of M2 macrophages with current anti-fibrotic medications or other immunological strategies, it is anticipated that a more powerful synergistic effect can be achieved, leading to enhanced therapeutic outcomes for patients. A successful approach is to integrate M2 macrophage therapy with antifibrotic medications, decreasing fibrotic tissue and enhancing tissue regeneration more efficiently.^99^

 Furthermore, due to the frequent occurrence of chronic inflammation before LF, the utilization of M2 macrophage therapy in conjunction with anti-inflammatory substances like toll-like receptors (TLR)-2 or NF-κB inhibitors holds great potential as a viable strategy.^100^ This combination can inhibit inflammation while enhancing the regenerative function of M2 macrophages, potentially decreasing fibrosis and enhancing the overall health of the liver. This method enables a concurrent decrease in the inflammatory process and expedited and more effective tissue regeneration.

 In addition, the research is also focused on combination therapy involving M2 macrophages and stem cells, particularly mesenchymal stem cells (MSCs). MSCs can regulate the immune system and facilitate tissue regeneration. By combining these two therapies, it is anticipated that there will be a synergistic impact on lowering fibrosis by promoting the regeneration of hepatocytes and reducing the synthesis of ECM.^101^ Furthermore, the utilization of PPAR-γ agonists, which have demonstrated the ability to shift macrophages towards the M2 phenotype, can augment the antifibrotic impact of this treatment,^102^ making it a more comprehensive and efficacious approach in the management of LF.

## Conclusion

 LF results from prolonged inflammatory responses to factors such as viral infections, excessive alcohol intake, and metabolic disorders. M2 macrophages, with their anti-inflammatory and reparative properties, present a promising target for immunotherapy in LF. They have a key function in modulating key signaling pathways, which suppress pro-inflammatory responses and facilitate tissue regeneration. However, challenges such as individual variability in treatment response and the risk of exacerbating fibrosis necessitate careful regulation of M2 macrophage activity. To optimize the safety and efficacy of M2 macrophage-based therapies, further research and technological advancements are essential. Overall, targeting M2 macrophages offers a novel approach to improving outcomes in LF treatment.

## Competing Interests

 The authors declare no conflict of interest.

## Ethical Approval

 Not applicable.
